# *Thermococcus bergensis* sp. nov., a Novel Hyperthermophilic Starch-Degrading Archaeon

**DOI:** 10.3390/biology10050387

**Published:** 2021-04-29

**Authors:** Nils-Kåre Birkeland, Boyke Bunk, Cathrin Spröer, Hans-Peter Klenk, Peter Schönheit

**Affiliations:** 1 Department of Biological Sciences, University of Bergen, N-5020 Bergen, Norway; 2 Department Bioinformatics and Databases, Leibniz Institute DSMZ, D-38124 Braunschweig, Germany; boyke.bunk@dsmz.de (B.B.); ckc@dsmz.de (C.S.); 3 School of Natural and Environmental Sciences, Newcastle University, Newcastle Upon Tyne NE1 7RU, UK; hans-peter.klenk@ncl.ac.uk; 4 Institut für Allgemeine Mikrobiologie, Christian-Albrechts-Universität Kiel, D-24118 Kiel, Germany; peter.schoenheit@ifam.uni-kiel.de

**Keywords:** archaea, hyperthermophiles, oil-well, genome, deep biosphere

## Abstract

**Simple Summary:**

Hyperthermophiles grow optimally above 80 °C and include mostly microorganisms belonging to the Archaea domain and are thriving in terrestrial and seafloor geothermal vents as well as in subsurface environments. From an anaerobic hyperthermophilic mixed culture obtained from water produced from a deep and hot oil reservoir we isolated and characterized a starch-degrading strain. Based on phylogenomic analysis, the strain represents a novel hyper-thermophilic species belonging to genus *Thermococcus*, for which we propose the name *Thermococcus bergensis* sp. nov.

**Abstract:**

A novel hyperthermophilic archaeon, termed strain T7324^T^, was isolated from a mixed sulfate-reducing consortium recovered from hot water produced from a deep North Sea oil reservoir. The isolate is a strict anaerobic chemo-organotroph able to utilize yeast extract or starch as a carbon source. The genes for a number of sugar degradation enzymes and glutamate dehydrogenase previously attributed to the sulfate reducing strain of the consortium (*Archaeoglobus fulgidus* strain 7324) were identified in the nearly completed genome sequence. Sequence analysis of the 16S rRNA gene placed the strain in the *Thermococcus* genus, but with an average nucleotide identity that is less than 90% to its closest relatives. Phylogenomic treeing reconstructions placed the strain on a distinct lineage clearly separated from other *Thermococcus* spp. The results indicate that the strain T7324^T^ represents a novel species, for which the name *Thermococcus bergensis* sp. nov. is proposed. The type strain is T7324^T^ (=DSM 27149^T^ = KCTC 15808^T^).

## 1. Introduction

The Euryarchaeota order (Thermococcales) encompasses a diverse and widely distributed group of thermophilic and hyperthermophilic archaeal members isolated from geothermally heated environments [1]. The order contains three genera; *Thermococcus* [2], *Pyrococcus* [3] and *Palaeococcus* [4]. According to the list of prokaryotic names with standing in nomenclature (LPSN; http://lpsn.dsmz.de, accessed on 2 March 2021), the genus *Thermococcus* contains 33 validly described species, all being organotrophic and strictly anaerobic hyperthermophiles. They grow preferentially with proteinaceous compounds such as yeast extract or peptone and carbohydrates. Growth temperatures range from 50 to over 100 °C. Based on their DNA G + C content, *Thermococcus* spp. can be divided into a low G + C content group (38 to 47 mol%) and a high G + C group (50 to 58 mol%) [5]. Most *Thermococcus* members have a marine origin, from deep hydrothermal vents or geothermally heated seashore environments. Two species, *Thermococcus zilligii* and *Thermococcus waitotapuensis*, originate from terrestrial fresh-water hot springs [6,7] while one species, *Thermococcus sibiricus*, originates from a high-temperature deep oil reservoir [8]. 

Deep petroleum reservoirs represent extremophilic subsurface environments with a diversity of anaerobic thermophilic micro-organisms [9,10,11] (e.g., thermophilic sulphate reducers, Thermotogales species, methanogens and even unique deep subsurface thermophiles such as *Thermovirga* and *Petrotoga* [12,13]). The hyperthermophilic archaeal sulphate reducer, *Archaeoglobus fulgidus*, was initially isolated from a shallow hot vent at Vulcano island, Italy [14], and subsequently isolated from a deep North Sea oil field [15]. 

The North Sea culture deposited at DSMZ (DSM 8774) in 1994 grew with lactate as a carbon and energy source under sulfate-reducing conditions but was later found to also grow on starch under sulfate-reducing conditions using a modified Embden–Meyerhof pathway [16]. However, genome sequencing later revealed a contamination with a *Thermococcus* strain, which apparently was responsible for the starch degradation [17]. Here we describe a novel *Thermococcus* species that originated from the North Sea *A. fulgidus* culture, which was believed to be a pure culture at the time of deposition. 

## 2. Materials and Methods

### 2.1. Isolation and Cultivation

An active culture of DSM 8774 was obtained from DSMZ and cultivated anaerobically in 50 mL serum flasks under nitrogen with lactate and sulfate at 80 °C as described in [15]. To isolate the *Thermococcus* strain, active cultures were transferred to an anaerobic lactate-free medium containing the following components (L^−1^ distilled water): 18 g NaCl, 2 g MgCl_2_∙6H_2_O, 0.32 g KCl, 0.14 g CaCl_2_∙2H_2_O, 0.11 g K_2_HPO_4_∙3H_2_O, 0.02 g KH_2_PO_4_, 0.5 mL 0.2% resazurin, 1 mL of a stock solution of 0.2% (NH_4_)_2_Fe(SO_4_)_2_∙6H_2_O and 10 mL of a stock solution containing 1.5 g Titriplex I, 0.5 g MnSO_4_∙2H_2_O, 0.1 g CoCl_2_∙6H_2_O, 0.1 g ZnSO_4_∙7H_2_O, 0.01 g CuSO_4_∙5H_2_O, 0.01 g H_3_BO_3_, 0.01 g Na_2_MoO_4_∙2H_2_O and 0.2 g NiSO_4_∙6H_2_O per litre. After autoclaving, the medium was flushed with N_2_/CO_2_ at a 9:1 ratio, 30 mL 1M NaHCO_3_, 4 mL 0.5 M Na_2_S and 10 mL vitamin solution [18] were added and the pH was adjusted to 6.5. Following its transfer to 50 mL or 20 mL serum flasks, the medium was supplemented with yeast extract and peptone to 0.1% final concentration. The culture was transferred 10 times using the above medium, followed by two dilutions to extinction. Subsequent cultivation was performed in a marine *Thermococcus* medium (DSMZ medium 760 (http://www.dsmz.de/microorganisms/medium/pdf/DSMZ_Medium760.pdf, accessed on 2 March 2021) under nitrogen at pH 7.0 and 80 °C. 

### 2.2. Sequencing and Bioinformatics

For initial phylogenetic characterization, genomic DNA was extracted from cells grown in the medium used for isolation as described above using a modification of the cetyl trimethylammonium bromide method [19]. The partial 16S rRNA gene was amplified with general archaeal 16S rRNA primers (21F, 5′-TTCCGGTTGATCCYGCCGGA-3′; 958R, 5′-YCCGGCGTTGAMTCCAATT-3′) and sequenced as described in [20].

For genome sequencing, DNA was isolated using a JetFlex Genomic DNA Isolation Kit (ThermoFisher Scientific). SMRTbell template library was prepared according to the instructions from PacificBiosciences, Menlo Park, CA, USA, following the procedure and checklist—20 kb Template Preparation using a BluePippin Size-Selection System. Briefly, for preparation of 15kb libraries 5µg genomic DNA were end-repaired and ligated overnight to hairpin adapters applying components from the DNA/Polymerase Binding Kit P6 from Pacific Biosciences, Menlo Park, CA, USA. Reactions were carried out according to the manufacturer’s instructions. BluePippin Size-Selection was performed according to the manufacturer´s instructions (Sage Science, Beverly, MA, USA). Conditions for the annealing of sequencing primers and the binding of polymerase to purified SMRTbell template were assessed with the calculator in RS Remote, PacificBiosciences, Menlo Park, CA, USA. SMRT sequencing was carried out on the PacBio RSII (PacificBiosciences, Menlo Park, CA, USA), taking a 240-min movie on two SMRTcells, which resulted in 101,716 post-filtered reads with a mean read length of 4947 bp.

Long read genome assembly was performed with the “RS_HGAP_Assembly.3” protocol included in SMRTPortal version 2.3.0 using default parameters (with the exception of the target genome size, which was increased to 10 Mbp). Assembly resulted in 61 contigs, from which only five revealed the expected coverage values (>130×). The draft genome was annotated using the NCBI prokaryotic genome annotation pipeline (PGAP) (https://www.ncbi.nlm.nih.gov/genome/annotation_prok, accessed on 2 March 2021) and RAST (http://rast.nmpdr.org/, accessed on 2 March 2021). Genome completeness was assessed using CheckM [21]. Pairwise average nucleotide identity (ANI) values and in silico genome sequence similarities (dDDH) were determined using the ANI calculator (http://enve-omics.ce.gatech.edu/ani/, accessed on 2 March 2021) and the genome-to-genome distance calculator available at DSMZ (http://ggdc.dsmz.de/, accessed on 2 March 2021).

## 3. Results and Discussion

**Figure 1:**
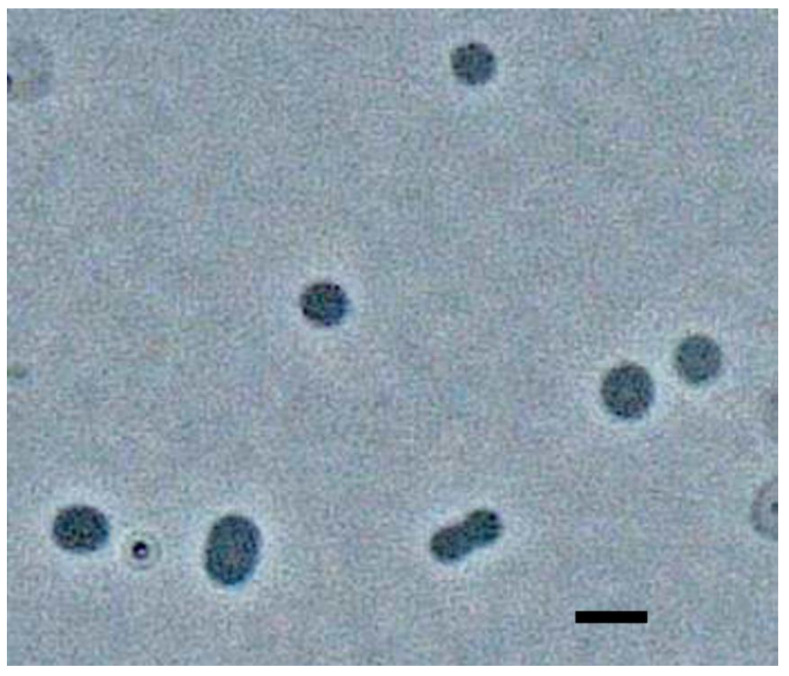
Phase-contrast microscopy image of strain T7324. Scale bar: 2 µm. The photo was taken at the.Leibniz-Institute DSMZ GmbH, Department Microbiology.

*A. fulgidus* depends on sulfate as an electron acceptor for anaerobic respiration and preferentially uses inorganic nitrogen sources and lactate as nitrogen and carbon sources, respectively, while thermococci are fermentative organisms that preferentially grow on complex media containing polysaccharides or proteinaceous compounds. To isolate the *Thermococcus* organism previously detected in this sulfate-reducing co-culture [17], the culture was transferred to the lactate-free and low-sulfate/ammonium medium described above and subjected to dilutions to extinction to enrich and isolate the *Thermococcus* organism. Following 10 culture transfers and two 10-fold dilution series, an apparent pure culture of coccoid to oblong cells with the typical morphology of *Thermococcus* members was obtained (Figure 1), with cell sizes ranging from 1–2 µm. Sequencing of the amplified 16S rRNA gene using universal archaeal PCR primers yielded a nearly complete sequence identical to that of *Thermococcus litoralis* DSM 5473 (NR_121707). The isolate, designated strain T7324^T^, was unable to grow using lactate and sulfate as an electron donor and electron acceptor, respectively. PacBio sequencing and assembly yielded five contigs with a base coverage of 137× and a size range of 0.85 to 1.2 Mb (Table 1). The total draft genome size was 2,077,832 bp (comparable with that of its closest relatives, *T. litoralis* (2,215,172 bases; CP006670.1) and *T. kodakarensis* (2,088,737 bp; NC_006624)). The genome completeness was estimated to be 99.01%. Overall genome sequence comparisons revealed an ANI value between *T. litoralis* and strain T7324^T^ of 87.9% and an in silico DNA-DNA hybridization value of 35.8%, which are significantly below the recommended species demarcation values of 98.7% [22] and 70%, respectively [23]. Based on the genome sequence difference, T7324^T^ therefore constitutes a separate species, for which we propose the name *Thermococcus bergensis* (ber.gen′sis N.L. masc. adj. bergensis referring to Bergen, the Norwegian city where the strain was isolated).

**Figure 2:**
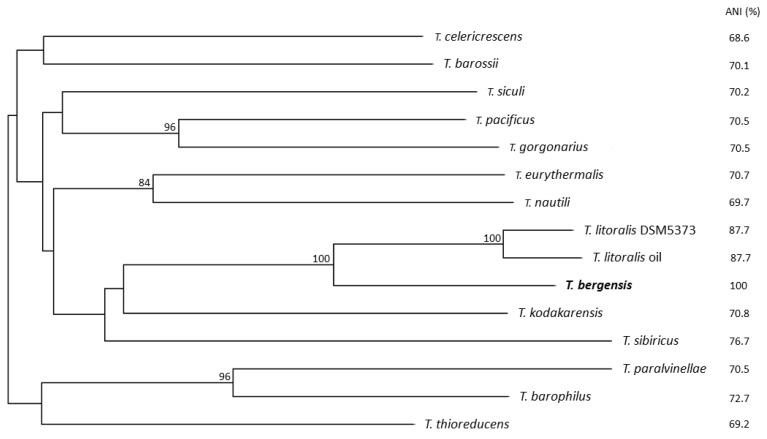
Phylogenomic tree of *T. bergensis* and other related *Thermococcus* type strains. The tree was inferred with FastME 2.1.6.1 [24] from genome blast distance phylogeny (GBDP) distances calculated from genome sequences using the TYGS server (https://tygs.dsmz.de, accessed on 2 March 2021) [22]. The branch lengths are scaled in terms of GBDP distance formula d5. The numbers above branches are GBDP pseudo-bootstrap support values >60% from 100 replications with an average branch support of 61.2%. The tree was rooted at the midpoint [25]. ANI values against *T. bergensis* calculated with OrthoANI (https://help.ezbiocloud.net/orthoani-genomic-similarity/, accessed on 2 March 2021) are shown to the right. Genome sequence accession numbers are as follows: *T. barophilus*, NC_014804.1; *T. barossii*, CP015101.1; *T. bergensis* 7324, JABFNK000000000; *T. celericrescens,* GCA_001484195.1; *T. eurythermalis,* GCA_000769655.1; *T. gorgonarius*, CP014855.1; *T. kodakarensis*, NC_006624.1; *T. litoralis* DSM 5473, CP006670.1; *T. litoralis* oil, GCA_900064395.1; *T. nautili*, CP007264.1; *T. pacificus*, CP015102.1; *T. paralvinellae*, NZ_CP006965.1; *T. siculi*, CP015103.1; *T. sibiricus*, CP001463.1; *T. thioreducens*, CP015105.1.

Another close genome neighbour of strain 7324, termed *Thermococcus litoralis* strain oil (NZ_FJMQ00000000), was identified after further searches in the NCBI nucleotide database. This strain is the closest relative of strain T7324^T^ based on overall genome similarity and also originates from a deep subsurface oil reservoir, but the two share an ANI value of only 89.85% and clearly represent different species. A phylogenomic tree including the closest relatives of strain T7324^T^ shows a distinct branching of *T. bergensis* from all the other species/strains with 100% bootstrap support and highly significant pairwise ANI values (Figure 2).

**Figure 3:**
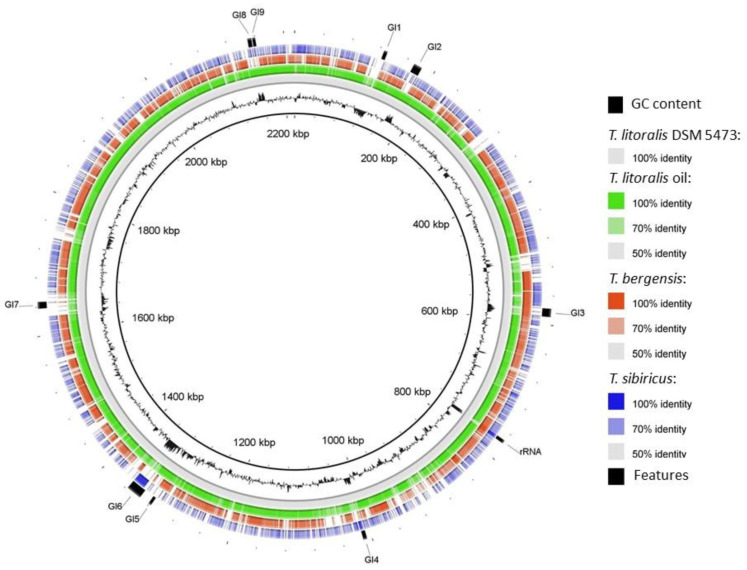
Circular BLAST ring image generator (BRIG) [26] representation of the *T. bergensis* T7324^T^ genome as compared with four representative *Thermococcus* species/strains using *T. litoralis* DSM5473 as a reference. Genomic islands (GI) were determined by SIGI-HMM implemented in IslandViewer 4 (https://www.pathogenomics.sfu.ca/islandviewer/, accessed on 2 March 2021). Ring colour codes are indicated to the right. The black (innermost) ring indicates the distribution of GC content of *T. litoralis* DSM5473. Genome sequence accession numbers are the same as in Figure 2.

The *T. bergensis* genome contained a single 16S/23S rRNA operon with two separately coded 5S rRNA genes, as is also the case for *T. litoralis* DSM 5473 (Table 1). The two strains differ by 1 mole% G + C, also indicating genetic diversification. Both strains contain a large number of CRISPR arrays but differ significantly in the number of spacers (Table 1). A simple genome comparison using the BLAST ring image generator (BRIG) with the complete *T. litoralis* DSM 5473 genome as reference revealed a number of non-homologous regions of various lengths, some of which corresponds to strongly GC biased genomic islands (Figure 3). These genomic islands possess a total of 200 predicted open reading frames, including 120 hypothetical genes and 18 genes annotated as membrane transport functions. As expected, the *T. litoralis* strain oil is significantly more similar to strain DSM 5473, while *T. sibiricus*, another *Thermococcus* oil reservoir isolate, shares with *T. bergensis* a number of non-homologous regions as compared to the strain DSM 5473 genome.

Based on the N-terminal sequences, all the genes encoding the previously purified thermostable enzymes for sugar degradation of the mixed culture, cyclodextrin gluconotransferase, maltodextrin–phosphorylase, phosphoglucomutase, ADP-dependent glucokinase, ADP-specific phosphofructokinase and pyruvate kinase were identified in the *T. bergensis* draft genome (Table 2), confirming the proposed sugar degradation pathway via cyclodextrins in this organism [27]. Thus, the growth and degradation of the starch and cyclodextrin of the mixed culture can be attributed to the *Thermococcus* strain of the mixed culture, which was enriched in comparison to the *A. fulgidus* strain during growth on sugars. Additionally, the gene for glutamate dehydrogenase previously purified and characterized from cells grown on lactate plus sulfate with yeast extract supplement [28] was identified (Table 2). Furthermore, the enzyme glyceraldehyde-3-phosphate ferredoxin oxidoreductase (GAPOR) was purified to homogeneity from a starch-grown culture of “strain 7324”. The purification consisted of four steps involving hydrophobic interaction and anion exchange chromatography on phenyl sepharose and Q sepharose columns, respectively, followed by gel filtration on Superdex 200 [29]. The gene encoding GAPOR was identified in the *T. bergensis* genome (Table 2) based on its N-terminal sequence, MRFSVLKINLNEKKVX(X)EVFEREXV. GAPOR from *T. bergensis* is a 66 kDa monomeric protein that contains 0.5 mol tungsten/mol but not molybdenum. The enzyme showed an optimum temperature above 80 °C and a high thermostability up to 100 °C [29]. GAPOR catalysed the oxidation of glyceraldehyde-3-phosphate (GAP) with benzyl viologen (BV) with apparent *V*_max_ and *K*_m_ values of 60 U/mg and 5 µM for GAP, and 73 U/min and 4 mM for BV, respectively, at 50 °C and pH 7.8. 

*T. bergensis* T7324 and A. *fulgidus* DSM 8774 have most likely constituted a co-culture since it was recovered from hot oil-field water in 1991 and deposited at DSMZ. The initial characterization did not include a phylogenetic analysis via sequencing of the 16S rRNA gene, and since the culture was routinely grown and transferred in a mineral medium containing only lactate as carbon and energy source with sulfate as electron acceptor, it was presumed to be a pure *A. fulgidus* sulfate-reducing culture. The addition of elevated amounts of yeast extract [28] or starch [16] apparently enriched for the *T. bergensis* strain. Despite numerous trials to isolate *A. fulgidus* DSM 8774 from this co-culture by dilution to extinction and the picking of colonies from gelrite-based anaerobic shake-tubes with lactate plus sulfate medium, the establishment of a pure culture has not been successful (Birkeland, unpublished), indicating an obligate syntrophic relationship between the two strains. Furthermore, although growing in isolation, *T. bergensis* grows much better as part of the co-culture, for reasons still not understood. 

## 4. Concluding Remarks

The *A. fulgidus* DSM 8774 culture was found to be contaminated with a novel *Thermococcus* species, termed *T. bergensis* T7324, which was isolated and genome sequenced. The previously reported growth of the DSM 8874 culture on starch is now shown to be attributed to the *T. bergensis* partner of this consortium. There is an obligate syntrophic relationship between the two strains, as *A. fulgidus* 7324 has not been able to grow in pure culture. The molecular basis for this syntrophy has not been resolved.

## Figures and Tables

**Table 1:** Genome statistics of *T. bergensis* T7324^T^ and *T. litoralis* DSM 5473^T^.

	*T. bergensis* T7324^T^	*T. litoralis* DSM 5473^T^
GenBank accession	JABFNK000000000	CP006670.1
Number of contigs	5 (draft)	1 (complete)
Number of base pairs	2,077,832	2,215,172
GC%	44.1	43.1
Number of rRNAs	2, 1, 1 (5S, 16S, 23S)	2, 1, 1 (5S, 16S, 23S)
Number of genes (total)	2370	2575
Number of CRISPRs	6 arrays, 129 spacers *	7 arrays, 251 spacers *

* Evidence level 4 as determined by CRISPRCasFinder (https://crisprcas.i2bc.paris-saclay.fr/, accessed on 2 March 2021).

**Table 2:** Enzymes previously purified from DSM 8774 cultures.

Enzyme	Reference	Locus Tag (in *T. bergensis*)
Cyclodextrin gluconotransferase (CGTase)	[27]	GQS78_01870
Maltodextrin–phosphorylase (MalP)^C^	[27]	GQS78_09095
Phosphoglucomutase (PGM)	[27]	GQS78_06950
ADP-dependent glucokinase	[30]	GQS78_06970
ADP-dependent phosphofructokinase	[31]	GQS78_11830
Pyruvate kinase	[32]	GQS78_00030
Glutamate dehydrogenase	[28]	GQS78_10575
Glyceraldehyde-3-phosphate ferredoxin oxidoreductase	[29]	GQS78_03205

## Data Availability

The *T. bergensis* DSM 8874 genome sequence is deposited in GenBank under accession number JABFNK000000000.
